# Perceptions and experiences of men and women towards acceptability and use of contraceptives in underserved areas of Karachi, Pakistan: a midline qualitative assessment of Sukh initiative, Karachi Pakistan

**DOI:** 10.1186/s12978-020-00946-3

**Published:** 2020-06-16

**Authors:** Sarah Saleem, Narjis Rizvi, Anam Shahil Feroz, Sayyeda Reza, Saleem Jessani, Farina Abrejo

**Affiliations:** The Aga Khan University–Department of Community Health Sciences, Stadium Road, PO Box 3500, Karachi, 74800 Pakistan

**Keywords:** Contraceptive use, Family planning, Married men and women, Pakistan, Sukh initiative

## Abstract

**Background:**

Family planning (FP) is an essential component of Sustainable Development Goals (SDG) and contributes directly to SDG targets 3.7 and 5.6. In Pakistan, contraceptive use has remained stagnant over the past 5 years. This change has been very slow when compared to the FP2020 pledge. The Sukh initiative project was conceived and implemented to alleviate these challenges by providing access to quality contraceptive methods in some underserved areas of Karachi, Pakistan. A qualitative study was conducted to understand the perceptions and experiences of men and women towards acceptability and contraceptive use.

**Methods:**

A qualitative study was conducted at ten Sukh stations located in four towns of Karachi. Focus group discussions (FGDs) were conducted with married women of reproductive age (MWRA) and married men who received FP services through the Sukh initiative. Study participants were purposively sampled for focus group discussions (FGDs). Interview data was manually transcribed and analyzed using thematic analysis.

**Results:**

A total of 20 FDGs (Men = 10 FGDs; MWRA = 10 FGDs) were conducted. Three overarching themes were identified: (I) Appropriateness and means to promote contraceptive use; (II) Equity and Accessibility to contraceptives; and (III) Perspective on available FP services. Generally, both men and women were informed about FP methods but women were more cognizant of FP information. The door to door services by community health workers in Sukh initiative areas was largely appreciated both by women and men as it has made the accessibility and availability of the information and services easy. Women suggested that the Sukh initiative should bring some strategies that can help men broaden their perspective towards FP. The study informed that the men feel left out from the FP programs. Therefore, male participants expressed keen interest in initiatives for men in their communities that would cater to their FP needs.

**Conclusions:**

This qualitative study provided a unique opportunity to understand the perceptions of men and women towards the phenomena of contraceptive use. The study identified the need for trained and qualified female and male healthcare providers and well-established health facilities alongside door-to-door services.

## Plain English summary

Contraceptive use helps couples and individuals realize their basic right to decide freely and responsibly if, when and how many children to have. Couples having fewer children can provide good education and health facilities to their children, as it gets affordable. The use of contraceptive methods has remained stagnant over the past 5 years (34% in the 2017–18 PDHS and 35% in the 2012–13 PDHS). This change has been slower when compared to the FP2020 pledge-55% CPR and 2.6 TFR by 2020. The Sukh initiative project was conceived to alleviate these challenges by providing access to quality contraceptive methods to promote the well-being of mothers and children and supporting the overall health and development of communities in some of the most underserved areas of Karachi, Pakistan. The study informed that generally, both men and women, were informed about FP methods but women were more cognizant of FP information. The Door to Door services by community health workers in Sukh initiative areas was largely appreciated both by women and men as it has made the accessibility and availability of the information and services easy. Women suggested that the Sukh initiative should bring some strategies that can help men broaden their perspective towards FP. The study informed that the men feel left out from the FP programs. Therefore, male participants expressed keen interest in initiatives for men in their communities that would cater to their FP needs.

## Background

Pakistan stands as the world’s seventh most populous nation [1]. According to the sixth population census, Pakistan’s population has surged to 207.8 million, having experienced a 57% increase since the last census in 1998 [2]. However, according to United Nations projections, the population of Pakistan will grow to 285 million by 2050 and will be ranked as the world’s fourth most populous country [3]. The slow pace in fertility decline is considered as one of the most important reasons for the huge projected increase in the population. There has been a steady decline in fertility rates over time, from 5.4 births per woman as reported in the 1990–91 Pakistan Demographic and Health Survey (PDHS) to 3.6 births per woman in the 2017–18 PDHS—a drop of about two births per woman in almost three decades [3]. However, this fertility rate was still considerably higher as compared to other countries of the South Asian region [4].

Family planning (FP) is an essential component of Sustainable Development Goals (SDG) and contributes directly to SDG targets 3.7 and 5.6. Investing in family planning is a development “best buy” that can improve outcomes in education, health, and wealth; help preserve the environment; protect the rights of and opportunities for women and girls, and increase food security for people [5]. Contraceptive use helps couples and individuals realize their basic right to decide freely and responsibly if, when and how many children to have. The growing use of contraceptive methods has resulted in not only improvements in health-related outcomes but also improvements in schooling and economic outcomes as well [1]. Couples having fewer children can provide good education and health facilities to their children, as it gets affordable. However, according to the Pakistan Demographic and Health Survey (PDHS) 2017–2018, 34% of currently married women use a method of family planning, with 25% using a modern method and 9% using a traditional method. Among currently married women, the most popular modern methods are the male condom and female sterilization (each used by 9%) [3]. There has been a slight decline in unmet need for family planning, from 20% in 2012–13 to 17% in 2017–18. However, the use of modern methods and the percentage of women with demand satisfied with modern methods have remained largely unchanged in the last 5 years [3]. The use of contraceptive methods has remained stagnant over the past 5 years (34% in the 2017–18 PDHS and 35% in the 2012–13 PDHS). This change has been slower when compared to the FP2020 pledge-55% CPR and 2.6 TFR by 2020 [6].

The Sukh initiative project was conceived to alleviate these challenges by providing access to quality contraceptive methods to promote the well-being of mothers and children and supporting the overall health and development of communities in some of the most underserved areas of Karachi, Pakistan [7]. The project was managed and supervised by a Program Management Unit (PMU). PMU identified implementation partners having knowledge and experience in each of the designed approaches and strategies. The Department of Community Health Sciences (CHS), Aga Khan University (AKU) was engaged as a monitoring and evaluation partner to inform program, measure progress and assess performance and impact. Therefore, baseline and midline assessments were carried out by the Department of Community Health Sciences, Aga Khan University.

The overall aim of the project was to increase the use of modern contraceptives among married couples, to ensure freedom of choice to the couples, respecting the fulfillment of human rights. A combination of approaches and strategies were implemented to address supply and demand issues of FP use among married men and women. These included house-to-house visits by female Community Health Workers (CHWs) for family planning counseling, referral and replenishment of supplies; life skills-based education for the youth; telehealth services to push tele-messages on family planning; and enhancement of quality of services of Family Planning and Maternal Neonatal and Child Health among selected public and private facilities at ten Sukh stations. This midline qualitative assessment was carried out in sukh project to understand the acceptability and use of contraceptives by married men and women among ten underserved areas of Karachi. Aga Khan University collected quantitative and qualitative information at baseline and then at midterm of the project after 18 months of interventions by Sukh Initiative. We present a qualitative assessment of the acceptability and use of contraceptive methods by the communities.

## Methods

The Sukh Initiative emerged out of commitments made at the London Summit held in July 2012. It is a partnership between three private Foundations, the Aman Foundation, the Bill & Melinda Gates Foundation and the David and Lucile Packard Foundation. Sukh Initiative is a six-year program (2013–2019), with goal of increasing the use of modern contraceptives by 15 percentage points amongst one million underserved peri-urban population of Karachi city of Sindh, Pakistan. Sukh Initiative is committed to provide FP related information, counseling, supplies, referrals and quality services to women of reproductive age residing in selected communities, being implemented by a consortium of six national and international organizations [7]. To understand the acceptability and use of contraceptives by married men and women among ten underserved areas of Karachi, the midline qualitative assessment was carried out by CHS department of AKU.

### Study design and setting

We used an exploratory qualitative research design to collect data from the purposively sampled population. The study was conducted at ten Sukh stations located in four towns of Karachi including Korangi, Landhi, Bin Qasim and Malir. The ethnicity differed in all the four towns. People in Landhi and Bin Qasim town were more conservative, poor and less educated as compared to Malir and Korangi population. The ten Sukh stations included Shah latif town, Future colony, Mansehra colony, korangi near Bakri petrol pump, Hasrat mohani colony, Ittehad colony, Ghazi town, Umar marvi goth, Rehri goth and Mehran town.

### Study participants

Married women of reproductive age (MWRA) and men who received family planning services through Sukh initiative were purposively sampled for focus group discussions (FGDs) to explore the experiences and perceptions towards phenomena of contraceptive acceptability and use.

### Interview guide

Semi-structured interview guides for FGDs explored participants’ perceptions and experiences towards family planning (FP) services offered by CHWs under the Sukh initiative. The guide specifically explored participants’ perceptions regarding barriers related to FP use, acceptance for FP use, accessibility to FP services, preferred type of contraceptive methods, cost and quality of FP services and suggestions to promote contraception use in the community.

### Data collection

A free flow of information was encouraged, using probes from these discussions to obtain participants’ perceptions towards phenomena of contraceptive use and healthcare utilization. Interviews were conducted face-to-face in most commonly understood and spoken local language ‘Urdu’ and were audio-recorded with consent from study participants. Detailed field notes were also taken during each interview to capture non-verbal language and cues. FGDs lasted for 45 min to 1 h and consisted of 8–12 participants per group.

### Analysis

To analyze qualitative data, thematic analysis was used for the FGDs. Data was collected and analyzed through an iterative process and data collection was ceased once data saturation was achieved. Data saturation refers to the point in the research process when no new information is discovered in data analysis, and researchers can claim that they have collected enough data to achieve their research purpose [8]. First, the audio recordings were transcribed into Urdu language and then translated in to English Language. The transcriptions and field notes were read several times to develop an interpretation of the participants’ perception towards phenomena of contraceptive acceptability and use. Focus groups with men and MWRA were coded as one data set. Two investigators coded a subset of transcripts independently which were then combined to match codes, and an agreement was sought on a coding technique. The remaining transcripts were then independently coded according to the framework. Codes were formulated inductively from the transcripts. Coding discrepancies were discussed and resolved to reduce the researcher’s bias. Codes were then analyzed under major themes. Semi-structured interview guides were used to outline major themes. Final overarching themes were discussed and reviewed by both investigators. To ensure the credibility of the research, study data were triangulated by the data sources (men and MWRA), to compare alternative perspectives and assess any inconsistencies.

### Ethical considerations

Ethical approval for this study was obtained from the Aga Khan University Ethical Review Committee (AKU-ERC). Written informed consent was provided by all study participants. Informed consent included permission to audio record the interviews and use anonymized quotes.

## Results

In this qualitative study, twenty FGDs were conducted;10 with men and 10 with MWRA, between January 2017 and February 2017, to understand participants’ perceptions and experiences towards the acceptability of contraceptives and their use. FP services offered by CHWs under Sukh initiative (Table 1). The demographic characteristics of FGD participants, including MWRA and men, are presented in Table 2. Based on the data collection and thematic analyses, three overarching themes were identified: (I) Appropriateness and means to promote contraceptive use in catchment population of Sukh Initiative; (II) Equity and Accessibility to contraceptives in catchment population of Sukh Initiative; and (III) Perspective on available FP services in the area in relation to cost, and quality of service. These themes are presented below with illustrative quotes.

**Table 1:** Study Participants

Focus Group Discussions with MWRA and men at 10 Sukh stations		Total FGDs = 20; ***n*** = 201
**FGD no.**	**Sukh Station**	**FGDs with MWRA**
1	Shah latif town	11
2	Future colony (Landhi)	11
3	Mansehra colony (landhi)	10
4	korangi near Bakri petrol pump	12
5	Hasrat mohani colony korangi	12
6	Ittehad colony	9
7	Ghazi town malir	8
8	Umar marvi goth	11
9	Rehri goth	9
10	Mehran town	8
**FGD no.**	**Sukh Station**	**FGDs with men**
1	Future colony (Landhi)	12
2	Mansehra colony (landhi)	9
3	korangi near Bakri petrol pump	9
4	Hasrat mohani colony korangi	11
5	Ittehad colony	8
6	Ghazi town malir	10
7	Umar marvi goth	12
8	Shah latif town	8
9	Rehri goth	12
10	Mehran town	9

**Table 2:** Profile of FGDs participants

	Women	Men
**Age**	**n**	**n**
15–19	2	2
20–24	25	9
25–29	25	20
30–34	19	32
35–39	15	23
40–44	5	11
45–49	11	2
**Education**		
None	49	10
<Matric	25	37
Matric	13	38
Intermediate	10	7
Bachelor	4	4
Masters	1	3
**No. of Living Children**		
None	1	15
1–2	46	42
3–6	49	35
> 6	6	7

### Theme 1. Appropriateness and means to promote contraceptive use in catchment populations of Sukh initiative

Under the theme of appropriateness, the matters discussed were; source of and process to receive information on family planning methods, autonomy to use of family planning methods, change in the behavior and communities’ suggestions to promote contraception in the community.

#### Source and process of providing information

Both men and women, in all the FGDs, were aware of the family planning services offered by Sukh Initiative in their areas. This has received information on family planning mostly through Aman community health workers who visited their homes regularly. Few men mentioned receiving text messages on cell phones by Aman tele-health services and having met with male workers from the Sukh initiative to discuss spacing births and use of family planning methods. Those who had interaction with male motivators expressed their desire to receive family planning methods as well in addition to information.

*We receive information through text messages and phone calls. We get information from Aman male worker. Some men get messages on phone some don’t (Man, FGD- Korangi Town, Station 05).*

In all the focus group discussions except one, both men and women mentioned free camps being organized in their areas from where they get information on family planning and obtain contraceptives free of cost. Men and women also appreciated free transport services to visit a health facility by Sukh initiative.

The community men and women were well aware of other community resources providing information and services for family planning. They mentioned community Health center by Aga Khan University in one area, private clinics, Marie Stopes clinics, government-run family welfare centers, medical stores, NGOs offering FP services such as Green Star and HANDs, internet, hospitals and, general clinics and Lady Health Workers of government Primary Health care and family planning program. However, with the presence of Sukh initiative, they are not only receiving information but are also getting free of cost pills, condoms and injections at their doorsteps which is cost saving for them. Both men and women expressed that the presence of men and women workers of the Sukh initiative had created a platform for friends, family members and, spouses to discuss and exchange knowledge related to family planning methods and their use. Men and women both mentioned that the presence of Aman health workers in their areas had made the accessibility and availability of information and services easy for them. It should be noted that male workers were far less in numbers as compared to female community workers hired by Sukh Initiative.

*Attending meetings have raised awareness in us, which we never had before (MWRA, Bin Qasim Town, Station 03).*

In almost all the FGDs, men mentioned that female CHW of Sukh Initiative provides information mostly to their women and ask them to share this information with their husbands. Very few men mentioned CHWs talking to them directly. Men expressed their concerns about not having any facility especially for them to provide firsthand information to them on family planning methods.

*Make some system such as a center or a clinic where men and women can go and get some information on family planning, it does not look nice that CHWs come to our house again and again. (Men, Bin Qasim Town, Station 2).*

Men and women mentioned that they only get information through CHWs as there is no other source for them to get information or supplies. Men raised concerns about women not having the privacy at home to discuss methods or their issues comfortably as other family members are also present at the time of the CHW’s visit and women feel embarrassed to discuss openly.

Some men from Bin Qasim and Landhi town had conservative views for use of family planning methods and considered this against religion. Some counter-argued that parents are answerable to God and to their children for providing good food and education to children and with small families they can do it easily.

*There are many religious institutes in these communities that don’t support FP; this is the reason people don’t want to use a family planning method. (MWRA, Bin Qasim Town, Station 3).*

*Meetings with Sukh’s counselors and religious leaders have removed many of our misconceptions, and now we think positively about family planning and are also allowing our children to have polio drops. (Men, Landhi Town, Station 9).*

#### Autonomy to use FP methods

In conservative populations of Bin Qasim and Landhi Towns, different views were expressed by women for use of family planning methods; in some focus groups, women mentioned strong role of husbands in their decision-making for going out of the house or to use a family planning method and mentioned that having information on family planning methods would have no effect on its use, as their husbands do not approve use FP methods. While in other groups, women mentioned having conditional autonomy to go out of home and use FP methods in mutual agreement with husbands, while some use FP without husbands or anyone else’s knowledge. In some FGDs women mentioned that men would not want any protection for themselves but would not stop women to use methods to prevent pregnancy.

While some men mentioned that their women generally stay at home, don’t interact with neighbors and do not share any information with each other either. Men from Landhi area mentioned feeling embarrassed to discuss FP related matters in front of elders or younger family members. However, both men and women believed that the decision to use family planning methods rests with individual families and there were no communities or institutional pressures against its use.

‘We were initially not aware of the FP methods, now we are aware about women’s health and education. We know that spacing of two years after birth help mother to regain her health”.

FGD-Men Rehri Goth.

Men and women from Malir and Korangi, which are relatively liberal areas, were supportive of the use of family planning methods. Men said they have no restrictions on their women to use family planning methods. Some women mentioned using implants without the permission of their husbands and had faced no reaction from their husbands.

Regarding cultural barriers, men in almost all the focus group discussions denied such barriers and mentioned that education is becoming common and people have information. Women do consult their husbands or in some cases, men’s permission is sought and couples decide jointly about matters especially related to family planning and health. The role of other family members such as mother in law is declining to a large extent, and the nuclear family is becoming a norm.

#### Change in behavior/thinking

Men in almost all the FGDs mentioned that FP use is most beneficial to their children and family in terms of the provision of food and education. But now they are equally concerned about the health of their women due to repeated and closely placed pregnancies.

*Prior to this (CHWs giving awareness of FP to community), we did not think about the health of women but now we do and do spacing of 2 years. (Men, BinQasim Town, station 2).*

Some of the reasons for the change in behavior against large family size were related to low financial resources, increasing interest in educating children, and health concern of spouses. Through Sukh Initiative the accessibility and availability of knowledge, information and services had made the use of FP easier for both men and women. The openness in discussions and mutual transfer of information had increased, resulting in a positive attitude towards the use of family planning methods. Men and women both showed the desire for having separate sessions on family planning for unmarried boys and girls in privacy.

*The way you are guiding our married ladies about family planning, you can guide our unmarried ladies, so that in future they can plan their family better. (Men, Malir Town).*

### Theme 2. Equity and accessibility to contraceptives in catchment populations of Sukh initiative

#### Accessibility

As far as affordability of the commodities is concerned men and women both mentioned that some people can afford the cost of family planning methods and most cannot.

Men mentioned that in spite of other resources available in the area, they prefer community health worker of Sukh initiative for providing information and methods such as condoms, pills, and injections at homes and providing the transport, cost of IUCD, and Implant and their insertion charges free of cost through Sukh initiative.

*We used condoms even before Aman CHWS were here and would get them from family welfare centers, now we receive these at our homes free of cost. (Men, Bin Qasim Town, station 2).*

### Theme 3. Perspective on available FP services in the area in relation to cost and quality of services

#### Methods and preferences

Nearly in every FGD, men were of the opinion that the use of condoms is easy; it is free of side effects and easily available. Men mentioned that other methods such as IUCD and implants from the private sector are expensive and treatment of side effects gets even more expensive, and then there is added cost of transport. In addition, the value of time spent on waiting and travel was also mentioned as a barrier for use of FP methods.

Women voiced concerns about the affordability of the family planning methods. They mentioned that due to high living cost it gets difficult to make ends meet, so cheaper methods are preferred such as condoms and pills. Men mentioned that only 10% of women might be using injections, because of its cost. Condoms are cheap and easily available.

*Injection and condoms are cheaper and safer; the rest of the methods are too expensive. (Men, Korangi Town, Station 7).*

Women mentioned that in case they don’t get a method from CHW or from a family planning clinic in the area, then their men buy these from shops. Men said that in case of non-availability of FP methods from the clinics or CHWs they usually get these from the open market where the cost of the FP method is dependent on the duration of spacing they provide and also on quality, the service inclusive of IUCD is expensive, which many people cannot afford.

#### Quality of services- concerns

Women mentioned side effects related to injection, IUCD, and implant as the main deterrent for their use. The main side effects mentioned were feeling of general tiredness, breakthrough bleeding, bloating, amenorrhea, weight gain, low BP and weak eyesight. Women stated that in case of any side effects they discuss with Aman health worker, or visit government-run facility.

*The withdrawal method did not work for us and I had my daughter, so I switched to injections but my health got badly affected and I have stopped using any FP method. (Woman, Landhi Town, station 9).*

*I have an IUCD placed, for the last 2 months and I am having bleeding. The health care provider gives me 3–4 tablets to take and asks me to tolerate it. This is not solving my problem. (Woman Landhi Town, Station 9).*

It appeared that women want a long duration of spacing but are perplexed with their side effects, its related cost of management, difficulty in performing religious rituals and intolerant behavior of husbands for denial of sex. They raised their concerns about the inability of the health care providers (CHWs and facility staff) to advise them on side effects. In all the FGDs women mentioned that they were not appropriately counseled about the side effects and its management. The Sukh initiative health workers have limited knowledge and generally refer them to health care providers. Both men and women mentioned that CHWs also give referral slips, but when they reach a facility with this slip the story gets different, they ask for money for everything and at times misbehave too. Women had complaints about lack of professionalism especially, of government healthcare providers, about the high cost of the private sector and of doctors who place IUCD but don’t manage complications.

Women also had interesting perceptions about the treatment for side effects of a method. They dislike just receiving counseling or some tablets to stop bleeding and expect some dietary advice, an intravenous infusion with iron or just an infusion. It seems important to educate women, men, FP providers including CHWs about the side effects of FP methods and their management. The women should know about the side effects and their management of long-acting family planning methods.

Women and men both complained about the bad attitude of the staff at public health facilities. The bad attitude of government staff impels them to consult the private sector which is far more expensive but the attitude of the provider is good, surroundings are clean and the method of choice is available. However, they all were appreciative of the low or free of cost services from the government sector.

When asked about the *ideal FP services*, men and women responded by saying that services should be closer to their homes, there should be proper counseling, methods should be free of cost or of minimal cost.

*The facility should be nearby otherwise we are dependent on others; if it is near we can easily go there (MWRA, Malir Town, Station 10).*

Staff should talk to clients with respect, show caring attitude, be cordial and have a soft demeanor. Drugs should not be of the expiry date, there should be less waiting time and side effects should be treated free of cost. Ideal timings for facilities mentioned were in the afternoon from 3 to 4 p.m. when women had taken care of all the household chores.

Men mentioned that they are on job all day and are back in the late afternoon or early evening, they should have separate clinics in the evening, and men should talk to men. Women suggested having centers close to their houses where they can go themselves. Men, whereas mentioned having informational pamphlets that they can view in their own privacy.

## Discussion

This study explored the perception and experiences of married men and women of reproductive age towards the phenomena of contraceptive acceptability and use. Generally, women were more cognizant of family planning information type of FP methods, especially modern methods, sources of procurement, and FP centers of the area and were more favorable towards the use of contraceptive methods [9]. This is not surprising as the main target for use of family planning is women and Sukh initiative followed the same strategy. However, men had shown interest in learning more about family methods and this should be picked and addressed by the family program implementers in Pakistan if they plan to achieve the SDGs of 2020. Nonetheless, an interest to develop more understanding of FP methods, especially modern methods, was reiterated by almost all study participants. Unlike other studies [10, 11], almost all the participants considered family planning usage as an individual’s choice, which was not influenced by sociocultural contexts, or any religious or political institution. This may be due to accessibility and availability of the information and FP supplies at door step.

The study highlighted that the presence of lady community health workers and their home visits in Sukh initiative areas had made the accessibility and availability of the information and services easy for women and to a certain extent to men as well. These results are consistent with similar family planning studies, which suggests that the provision of FP services through CHWs is effective as it helps improve access and availability to contraceptives [12]. Both men and women recognized CHWs as the key agents for giving FP information to the Sukh catchment population; however, they were concerned about the privacy and confidentiality issues due to the presence of the other family members in the home during the CHWs visits. These findings are in agreement with previous research study, in which fear of breach of confidentiality was reported as the most important barrier that prevented women from seeking FP information and assistance from CHWs [13].

The study informed that in some towns the role of men continues to dominate in decision making, for family planning method used for women. While in others, women were more empowered for making their own decisions for contraceptive use. However, resistance for FP use on part of husbands’ or mother in law did not come up strongly as reported by other national studies [14]. Mustafa et al., documented that sociocultural pressures including in-laws’, husband’ and peer’ pressure on women discouraged contraceptive uptake by women [14]. Women in our study suggested that Sukh initiative should bring strategies that could help men broaden their perspective towards family planning to pave a way for a more autonomous life for MWRA. A similar study conducted in Sub-Saharan Africa also suggested that health education campaigns to improve the beliefs and attitudes of men are absolutely needed [4]. The study informed that the men feel left out from the FP programs. Male participants showed a strong desire for initiatives to cater to their FP needs with regard to information and services within their areas.

Despite the high willingness for use of FP methods, the desired family size reported by men and women was around 3–5 children. An encouraging finding in this aspect was the element of spacing between pregnancies that the community now feels necessary. It was also noted that there was a difference in opinion and attitude towards the use of FP methods amongst men and women of all towns. Landhi and Bin Qasim appeared to be more conservative while Korangi and Malir were comparatively more liberal in their attitudes towards acceptability and use of family planning methods. However, an overall finding was that the taboo so strongly associated with talking about FP was mitigated by the efforts of CHWs’ door to door home visits.

With regard to FP method preference, the condom was the most commonly used method, the choice of a method was dependent on ease of availability, and less known side effects. Amongst the modern contraceptives, the preferred choice amongst women included implants and IUCD. However, men and women both considered that modern methods (IUCD, Implant, injectable) and management of related side effects were costly, and was the reason for not opting for the modern methods of contraception.

Quality of services for their method of choice was associated with the duration of the protected period, management of side effects and above all the attitude of the health care provider. The main concerns were related to the impolite attitude of the facility staff of the public sector. Women raised concerns about the inability of the healthcare providers (CHWs and facility staff) to advise them on side effects and their management. Addressing concerns such as appropriate side effect management, access, ensuring the availability of trained and qualified female healthcare providers and quick and easy access to contraceptives will help meet the FP needs of women and couples [14].

The findings of this study should be treated cautiously to avoid generalization to areas that have different sociodemographic characteristics, due to limitations arising from the study design. Additionally, this study is conducted with the limited number of people and therefore it may not reflect the perceptions of the whole population in the catchment population. More importantly, the study documented the views and experiences of married men and women, thus their perceptions may vary from the opinions of unmarried girls and boys with different socioeconomic and demographic characteristics.

## Conclusion

This study provided insights into the perceptions of men and women towards contraceptive use under Sukh initiative. Generally, all study participants appreciated the presence of community health workers as it has made the accessibility and availability of the FP information and services easy for women and men to a certain extent. The study informed that the subject of FP has now become a topic of discussion amongst women gatherings, amongst men and amongst couples. Thus the existing pressures of community norms, religion and families are changing and paving way for small family norms. The study identified the need for trained and qualified female and male healthcare providers and well-established health facilities alongside door-to-door services.

## Data Availability

The datasets used and/or analyzed during the current study are available from the corresponding author on reasonable request.
